# Effectiveness of Individual-Based Strategies to Reduce Nurse Burnout: An Umbrella Review

**DOI:** 10.1155/2024/8544725

**Published:** 2024-04-23

**Authors:** Hsiang-Chin Hsu, Huan-Fang Lee, Hsuan-Man Hung, Yi-Lin Chen, Miaofen Yen, Hui-Ying Chiang, Lok-Hi Chow, Susan J. Fetzer, Pei-Fan Mu

**Affiliations:** ^1^Department of Emergency Medicine, National Cheng Kung University Hospital, College of Medicine, National Cheng Kung University, Tainan, Taiwan; ^2^Department of Emergency Medicine, School of Medicine, College of Medicine, National Cheng Kung University, Tainan, Taiwan; ^3^School of Medicine, College of Medicine, National Cheng Kung University, Tainan, Taiwan; ^4^Department of Nursing, College of Medicine, National Cheng Kung University, Tainan, Taiwan; ^5^Taiwan Holistic Care Evidence Implementation Center: A JBI Affiliation Center, Taichung, Taiwan; ^6^Department of Nursing, Fooyin University, Kaohsiung, Taiwan; ^7^Nursing Department, National Cheng Kung University Hospital, College of Medicine, Tainan, Taiwan; ^8^Nursing Department, Chi Mei Medical Center, Tainan, Taiwan; ^9^Department of Anaesthesiology, Taipei Veterans General Hospital, Taipei, Taiwan; ^10^Department of Nursing, College of Health and Human Services, University of New Hampshire, Durham, NH, USA; ^11^Institute of Clinical Nursing, National Yang Ming Chiao Tung University, Taipei, Taiwan

## Abstract

**Aims:**

This umbrella review aims to comprehensively synthesize and analyze the findings of available systematic reviews on the effectiveness of individual-based strategies for reducing nurse burnout occurring in hospital-based settings.

**Methods:**

Following JBI guidelines, an umbrella review was conducted to integrate the effectiveness of various strategies to reduce burnout. Systematic reviews were searched in the Embase, MEDLINE (Ovid), Cochrane Library, CINAHL (EBSCO), Scopus, and WOS databases. Inclusion criteria included studies published in any language from database inception to April 2023. Eligibility assessment involved two independent reviewers who evaluated titles, abstracts, and full texts. The systematic reviews were critically evaluated using JBI SUMARI. The results were narratively synthesized and grouped by strategy.

**Results:**

Eleven systematic reviews were included, covering the years 2012 to 2021. The appraisal tools varied, though all included reviews were of high quality. The strategies were categorized into three domains: mental health (51%), physical activities (26%), and professional competence (13%). The interventions most identified were mindfulness-based stress reduction for mental health, yoga for physical activities, and professional competence education. These individual-based strategies were shown to effectively eliminate emotional exhaustion (72.7%), depersonalization (44%), and occupational stress (78%) among nurses in hospital-based settings.

**Conclusion:**

Mental health, physical activities, and professional competence are strategies to reduce nurse burnout. Implementing these approaches in healthcare settings can improve emotional exhaustion, depersonalization, and occupational stress of nurses.

## 1. Introduction

Globally, nurse burnout is a critical issue impacting on the healthcare workforce which is reported as 11.23% of burnout symptoms [1] and continuously spreading out within the healthcare sector [2].

Indeed, nurse burnout is amplified during the COVID-19 pandemic, given their increased vulnerability to the virus and the multifaceted challenges encountered in providing care [3, 4]. Nurses constitute a large proportion about 60% in hospitals, and an increasing number of nurses are required to assist critical and general patient care during the COVID-19 pandemic [5].

Burnout in nurses has numerous detrimental effects on individuals, organizations, and patient care. It significantly affects the health and well-being of nurses by emotional exhaustion, depersonalization, and low personal accomplishment. These symptoms can have adverse consequences for nurses, including the development of physical and mental health problems such as depression and anxiety [6, 7]. Nurse burnout is linked to poor outcomes such as quality of care and patient satisfaction [6]. Burnout can be detrimental to patient care quality, leading to an increase in medical errors that compromise patient safety [8]. Addressing nurse burnout becomes imperative to uphold quality patient care and sustaining the healthcare system. Improving nursing burnout requires interventions at various levels, including national and organizational policy such as creating a positive work environment and healthy workforce strategies, as well as individual-based approaches. While national and organizational strategies to reduce burnout are generally directed towards the majority, individual-based strategies are needed to be designed for nurses to choose according to their preference.

Numerous interventions have been suggested to assist nurses in practicing self-care with the aim of mitigating or preventing burnout and various individual health-related outcomes [9–13]. For example, physical activities such as yoga, Qigong, and Tai Chi have been proposed to improve sleep quality and alleviate post-shift stress [10]. Emotion-focused tactics and psychosocial programs have been implemented to enhance mental health and prevent burnout [12]. A variety of mindfulness-based interventions (MBSR) have been advocated to enhance nurse well-being. These interventions have shown positive impacts on sleep quality, anxiety, depression, and overall resilience [13–15]. Interventions such as team-based training, communication skills enhancement, cognitive coping mechanisms, and problem-solving techniques have demonstrated efficacy in reducing nurse burnout and maintaining effectiveness [9, 11]. Multicomponent interventions have also positively affected physical and mental health and job satisfaction [12].

Recent research on nursing burnout has increasingly focused on multifaceted interventions with promising potential. These interventions often combined physical or psychological methods, yet researchers are interested in wide range of outcome indicators [16, 17]. Numerous original studies employing different approaches to reduce burnout have yielded varying results due to differences in study design and implementation [17, 18]. Systemic review and meta-analysis on the topic of burnout have emerged prominently since the 1990s and have been essential to integrate the best evidence while evaluating research biases [19]. However, differing criteria for research inclusion and exclusion, search terms, timeframes, language, and type of article have contributed to varied outcomes and interpretations on the efficacy of nurse burnout strategies. The increasing number of such reviews can be overwhelming for those seeking clinical application. Furthermore, while numerous interventions are frequently used, their comprehensive evaluation in many meta-analyses remains lacking [20].

An umbrella review, alternatively known as an overview of reviews, represents a unique literature review format that aggregates findings from multiple systematic reviews or meta-analyses on a specific subject [21]. Unlike traditional systematic reviews that examine primary studies, an umbrella review analyzes evidence from existing reviews to provide a more comprehensive overview of the research area. This method is particularly apt for a comprehensive synthesis of varied strategies and outcomes across a multitude of reviews, providing a more integrated understanding of effective interventions in a thoroughly researched domain [20, 21]. By compiling data from various reviews, an umbrella review yields clearer, more substantial, and elevated insights into the efficacy of individual-based strategies for mitigating burnout among nurses. This type of review facilitates a thorough assessment of current evidence, pinpointing both consistencies and discrepancies in the findings [22]. Therefore, the objective of this umbrella review is to comprehensively synthesize and analyze selected systematic reviews which have evaluated the effectiveness of individual strategies implemented to reduce burnout among nurses.

## 2. Methods

This umbrella review aims to synthesize the impact of individual-based strategies on reducing nurse burnout within hospital-based settings. The methodology adhered to the guidelines developed by the Joanna Briggs Institute [23] and the Preferred Reporting Items for Systematic Reviews and Meta-Analyses (PRISMA) reporting standards. The umbrella review was registered in the PROSPERO system (Number: CRD42022330618).

### 2.1. Search Strategy

Six databases, from inception to April 2023 were searched: Embase, MEDLINE (Ovid), Cochrane Library, CINAHL (EBSCO), Scopus, and WOS. The PICO framework guided this umbrella review with the question: Are individual-based strategies effective in reducing burnout among front-line nurses in hospital settings? Language was not restricted. The MeSH and free-text terms related to individual-related strategies for burnout in nurses were searched. The syntax was listed as [(nurse^*∗*^ OR (staff^*∗*^ OR employee^*∗*^ OR officer^*∗*^ OR personnel^*∗*^ OR practitioner^*∗*^ OR profess^*∗*^ OR provider^*∗*^ OR specialist^*∗*^ OR worker^*∗*^) NEAR/6 (nurs^*∗*^ OR health^*∗*^ OR hospital^*∗*^ OR medical))] AND [(burnout^*∗*^ OR “burn out^*∗*^” OR exhaustion^*∗*^ OR (extreme^*∗*^ ADJ 4 fatigue^*∗*^))]. The SR filter formula used the BMJ Best Practice syntax (https://bestpractice.bmj.com/info/toolkit/learn-ebm/study-design-search-filters/).

#### 2.1.1. Inclusion and Exclusion Criteria

The selection process applied the following inclusion criteria: (1) studies specifically targeting nurses, (2) interventions that were individual-based and aimed at reducing burnout, (3) primary outcomes related to burnout and their dimensions, such as emotional exhaustion, depersonalization, and reduced personal accomplishment, (4) the context as hospital-based setting, and (5) type of systematic reviews was intervention-based. Studies were excluded if (1) participant data were combined with other healthcare disciplines, (2) no provided data to address the effectiveness in the systematic reviews, and (3) insufficient information to appraising methodologic quality.

#### 2.1.2. Selection of Articles

Articles meeting the inclusion criteria were uploaded to EndNote X9 (Clarivate Analytics, PA, USA) for article screening. Two independent reviewers assessed eligibility by titles and abstracts followed by full text review of eligible studies. Reasons for the exclusion of papers that did not meet the inclusion criteria were recorded. Any disagreements between the two reviewers were resolved through discussion with a third reviewer. After relevant studies were retrieved, the JBI system for the unified management, assessment, and review of information (JBI SUMARI) (JBI, Adelaide, Australia) was applied to integrate findings.

### 2.2. Quality Appraisal

The methodology's quality was evaluated using the JBI SUMARI's systematic review instruments, consisting of the JBI Critical Appraisal Checklist for Systematic Reviews and Research Syntheses (JBI CACSRRS) [24]. The appraisal of systematic reviews or meta-analyses is guided by 11 questions. The answers are rated as “yes,” “no,” “unclear,” “or not applicable.” Two independent reviewers used the instruments to appraise eligible studies. A third reviewer was consulted to facilitate discussion and resolve any issues.

### 2.3. Data Extraction

The JBI SUMARI was used to extract data from the included reviews. The information extracted included first author's name and country, published year, and review objectives. The details of included studies in each review were extracted and included the number of studies, number of included participants, country, study design, strategies, outcome measurement, and conclusions.

## 3. Results

### 3.1. Search Process

**Figure 1:**
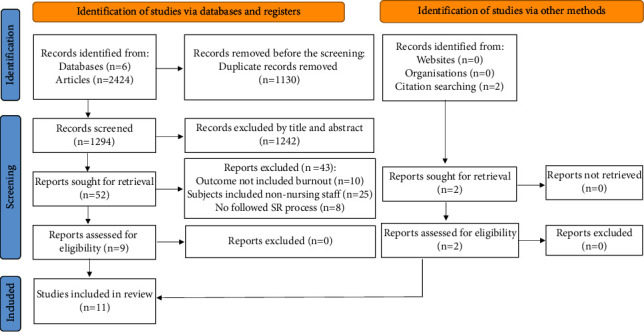
Preferred Reporting Items for Systematic Reviews and Meta-Analyses (PRISMA) flow diagram. From: Page MJ, McKenzie JE, Bossuyt PM, Boutron I, Hoffmann TC, Mulrow CD, et al. The PRISMA 2020 statement: an updated guideline for reporting systematic reviews. BMJ 2021; 372: n71. doi: 10.1136/bmj. n71.

A total of 2424 articles were retrieved from six databases. After removing duplicate records (*n* = 1130) and screening by title and abstract, 52 full-text articles were reviewed. Articles were excluded due to the following: the outcome did not include burnout (*n* = 10), subjects included non-nursing staff (*n* = 25), and the article did not present a systematic review (*n* = 8). Nine articles from six databases were retained. Two additional articles were obtained using a citation search. Eleven articles were included in the analysis (Figure 1).

### 3.2. Characteristics of Included Articles

The articles were published between 2012 and 2021, with two of the eleven systematic reviews including meta-analyses (Table 1). The number of studies synthesized with the systematic review ranged from 6 to 25, encompassing a total of 467 to 6,055 subjects. Researchers were represented from Germany (*n* = 4), Canada (*n* = 2), and one each from Taiwan, Korea, Australia, Malaysia, and Iran. The included studies in the review encompassed a global perspective, covering Europe (with countries such as the Netherlands, Spain, Italy, Germany, Ireland, the UK, Portugal, Denmark, Sweden, Norway, Greece, and France), the Americas (the USA, Canada, and Brazil), the Middle East (Iran and Israel), Asia (India, Japan, Turkey, China, Taiwan, Malaysia, Hong Kong, and Korea), and Oceania (Australia). The RCT was the most researched design within the reviews. The outcome indicators represented three categories: mental perception (e.g., stress, burnout, depression, and life satisfaction), physical symptoms (e.g., muscle pain and insomnia), and work-related (e.g., patient care and job satisfaction).

### 3.3. Quality Appraisal

The quality of the eleven included articles was evaluated using the JBI CACSRRS. In the eleven systematic reviews, 10 (90.9%) achieved a yes score on 8 of the 11 questions (Table 2). All articles met of 100% for questions 1, 7, 8, 10, and 11. Four questions (Q2, Q3, Q4, Q5, and Q6) were met between 64% and 91%. Question 9, “the likelihood of publication bias,” was answered for only one review due to the low number of studies in the other reviews. All eleven systematic reviews were included for qualitative integration.

### 3.4. Effectiveness of Strategies and Interventions

#### 3.4.1. Strategies and Interventions for Reducing Burnout

Among the 11 selected systematic reviews, a total of 145 studies were included. After removing duplicates, 131 remained, of which 64 studies discussed the three strategies for the outcome indicators of burnout or occupational stress. Within these 64 studies, the strategies employed included combinations in 6 studies (four mental health + physical activities and two mental health + professional education) and single strategies in 59 studies (25 for mental health, 24 for physical activities, 9 on professional education).

**Figure 2:**
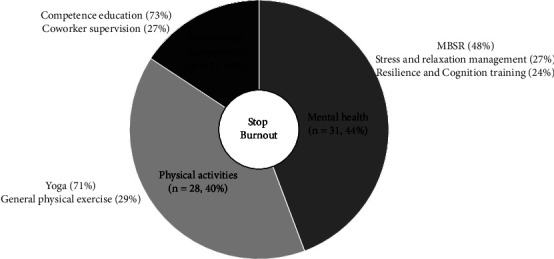
The strategies and interventions for reducing nurse burnout.

Among the 64 studies, the distribution of strategies applied was as follows: mental health (*n* = 31, 44%), physical activities (*n* = 28, 40%), and professional competences (*n* = 11, 16%). In the 31 studies focused on mental health strategies, three types of interventions were included: mindfulness-based stress reduction (MBSR), stress and relaxation management, and resilience and cognition training. Two studies combined two approaches (stress and relaxation management + resilience and cognition training; MBSR + resilience and cognition training). Thus, in these 31 mental health strategy studies, the distribution of the interventions was MBSR (48%), stress and relaxation management (27%), and resilience and cognition training (24%). In the 28 studies related to the strategies of physical activities, two types of interventions were yoga and general physical exercise, with their distribution being yoga (71%) and general physical exercise (29%). Among the 11 studies on professional competences strategies, two types of interventions were competence education (73%) and coworker supervision (27%) (Figure 2).

#### 3.4.2. Effectiveness of Interventions for Reducing Burnout

**Figure 3:**
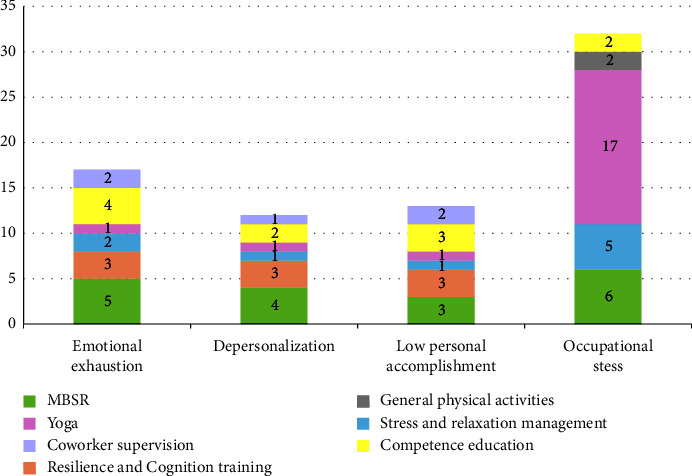
The effectiveness of interventions for reducing emotional exhaustion, depersonalization, low personal accomplishment, and occupational stress.

Among the 64 studies reviewed, 23 mentioned the effectiveness of interventions on emotional exhaustion (EE), with 17 (74%) reporting a significant reduction in EE. Among these 17 studies, the interventions most frequently effective in reducing EE included MBSR mentioned 5 times, competence education 4 times, resilience and cognition training 3 times, stress and relaxation management 2 times, coworker supervision 2 times, and yoga once. 19 studies addressed the impact of interventions on depersonalization (DP), with 11 (58%) reporting effective reductions. The most frequently effective measures for reducing DP included MBSR 4 times, resilience and cognition training 3 times, competence education 2 times, stress and relaxation management once, coworker supervision once, and yoga once. 20 studies discussed intervention effects on low personal accomplishment (LPA), with 10 (50%) achieving significant reductions. The interventions most effective in reducing LPA were MBSR, resilience and cognition training, and competence education, each mentioned 3 times, followed by coworker supervision 2 times, stress and relaxation management once, and yoga once. 42 studies examined the effectiveness of interventions on work stress, with 32 (76%) noting substantial stress reductions. The most frequently effective strategies to reduce work stress were yoga 17 times, MBSR 6 times, stress and relaxation management 5 times, competence education twice, and general physical activities twice (Figure 3).

## 4. Discussion

An umbrella review included a comprehensive evaluation of evidence derived from eleven systematic reviews of 131 different research studies focused on reducing nurse burnout. Strategies formed three main categories: mental health, physical activity, and professional competence. The interventions which positively contributed on burnout were MBSR, resilience and cognition training, and stress and relaxation as well as yoga in occupational stress.

### 4.1. Enhancing Mental Health

MBSR is the most frequent mental health strategy applied to reduce burnout based on the study findings. Nurses face a high workload and poor working conditions [29] and are at risk of developing psychological distress [30]. Many studies have documented the effectiveness of MBSR in reducing stress [13, 14]. For physical mechanism, MBSR practices can be crucial for the body's defense against infections and improving health [31, 32]. MBSR practices cultivate self-compassion, helping individuals to face adversity without succumbing to self-criticism or negative self-evaluation, which are key factors in burnout [33]. When conducted in group settings, mindfulness practices strengthen interpersonal connections among nurses, providing a network of emotional support that is vital for managing work-related stress and reducing the risk of burnout [34].

A typical MBSR program consists of two to three hours of instruction per week for eight weeks and requires regular practice to reap its full benefits. However, some factors should be considered for the individuals [35, 36]. (1) Time commitment: a typical MBSR program consists of two to three hours of instruction per week for eight weeks; it may be challenging for some individuals to commit this amount of time. (2) Requires practice: MBSR requires regular practice to reap its full benefits; a busy schedule may make this difficult for some individuals. (3) Not a substitute for professional help: the MBSR program should not be regarded as a substitute for professional medical or psychological care. In cases involving mental or physical illness, it is essential to seek professional assistance.

### 4.2. Increasing Physical Activity

Increasing physical activity was the second most frequently strategy for mediating nursing burnout, with yoga being the most common activity. Physical activity influences hormone levels, including the stress hormones. Maintaining physical activity provides a positive contribution to human psychoneuroimmunology and improved mental health [37]. The systematic review of Dutta et al. [38] described the physical and psychological benefits of yoga. However, yoga practitioners must be cautious of (1) musculoskeletal injuries: injuries to the musculoskeletal system are caused by the improper position, which can lead to muscle, bone, and joint problems [39]; (2) overstretching: an individual who overstretches their body or pushes it beyond its limits can lead to both pulled muscles and torn ligaments as a result of overstretching or pushing the body beyond its maximum ability [40]; and (3) physical exhaustion: for beginners or individuals with underlying health conditions, intense yoga sessions can sometimes result in physical exhaustion [41].

### 4.3. Improving Professional Competence

Professional competence was the third strategy, and professional competence education and coworker supervision were the important interventions in the current systematic reviews. The nurses' professional competence is reflected in their attitudes, knowledge, and psychosocial and psychomotor skills [42]. Nursing professional competence refers to the ability of nurses to demonstrate various abilities such as personal characteristics, professional attitudes, values, knowledge, and skills as they carry out their professional responsibilities [43]. Some researchers emphasized the significance of professional values on nursing competence and found the negative relationship between professional values and burnout [44, 45]. On the other hand, work-related stress occurs when people are expected to perform tasks beyond their abilities which requires coping mechanisms. Improving professional competence can provide confidence which can lead to a sense of mastery and control. Situational control reduces the stress associated with uncertainty and ambiguity for a decrease in burnout [45, 46]. Nurses possessing high levels of professional competence are typically well-equipped with the necessary knowledge, skills, and experience to adeptly handle challenging situations and workloads [47].

Additionally, nurses with higher levels of professional competence reported stronger relationships with their colleagues, likely because they are more likely to be seen as competent and trustworthy [48]. Many research studies have indicated that good interpersonal relationships are an important factor in combating burnout [49, 50]. Therefore, higher professional competence can positively contribute to good interpersonal relationships and self-confidence at work, which can then reduce stress and burnout.

### 4.4. Implications for Managers

The umbrella review highlighted the importance of manager facilitation in addressing nursing burnout through individual-based strategies. Key recommendations include routine assessment of burnout level, offering MBSR and yoga programs, fostering workplace social support networks, and organizing professional competence development programs.

First, regular assessment of the burnout level is necessary because the effectiveness of interventions would be ineffective after 6 months [11]. Managers can tailor the schedule for burnout assessments to align with the organization's culture and the individual characteristics of employees. This approach allows for a thorough evaluation of burnout levels, taking into account differences across various professional nursing tiers and considering significant occurrences like hospital accreditation or personal milestones. Second, managers can arrange the MBSR and physical activities such as yoga or general physical exercise training for nurses. Before the nurses practice the MBSR or yoga activities by themselves, well-trained instructors can provide comprehensive training and prevent the adverse events of MBSR and yoga. On the other hand, the instructors can suggest the appropriate period of MBSR or skill of yoga according to individual characteristics. Third, resilience improvement is also an important factor. Studies indicate that resilience can combat burnout [15, 26]. Health organizations need to improve the well-being of nurses and the managers can implement training courses such as resilience training to prevent the incidence of cumulative burnout [51]. Fourth, nurse leaders can mitigate the negative impact of burnout on their professional values by strengthening and improving their professional value education through seminars and nursing in-service education programs [52]. In addition, nurse managers can provide continuing education opportunities based on the working unit or level of professional capacity to enhance professional competence among nurses [53]. The competence improvement education can teach nurses to determine for themselves how to handle their problems and how to improve their situation through meaningful dialog and engagement with nurse leaders concerning their work-life issues. Finally, the peer support and supervision system is necessary. A network fosters a sense of belonging and security, enhancing individuals' ability to cope with stress and burnout. Designing leisure activities for nurses can contribute to interpersonal relationships and decrease stress [54].

## 5. Strengths and Limitations

An umbrella review systematically compiled and synthesized evidence on individual-based strategies for reducing stress and burnout among nurses in hospital-based settings. The review marks a significant step in collating evidence and identifying the research on the most frequently used strategies. The review was limited by only including published systematic reviews and omitted grey literature or unpublished studies. Overlapping sources across the systematic reviews led to inconsistent outcomes and may have rendered burnout metrics inaccurate. Additional mental health indicators, such as anxiety or depression, may have confounded burnout findings. The umbrella review revealed that while numerous interventions are commonly used, many have not been thoroughly tested. More research is needed for in-depth analysis of these interventions.

## 6. Conclusion

Strategies for reducing nurse burnout are focused on mental health, physical activity, and professional competence. Nurses can adopt personal preference strategies and self-help interventions to reduce burnout.

## Figures and Tables

**Table 1:** Key characteristics of systematic reviews in the umbrella review (*n* = 11).

First author/year/country	No. of included studies (year of publication)	Total no. of subjects	*n*/countries represented	*n*/study designs represented	*n*/strategies	Outcomes	Conclusions
Stuber/2021/Germany	7 (1994–2018)	1104	4 USA 1 China 1 UK 1 Germany	1 RCT 3 Cohort 3 CCT	2 Stress management and coping 2 Professional enhancement 2 Communication and conflict management 1 Problem-solving	↑ Work atmosphere ↑ Personal competences ↑ Work satisfaction ↓ Psychological strain ↓ Insomnia ↓ Emotional exhaustion	Leadership intervention can maintain or foster mental health among nurses

Jung/2021/Korea	17 (1993–2020)	1430	4 China 3 US 2 Taiwan 2 Japan 1 Korea 1 Greece 1 Turkey 1 France 1 Malaysia 1 Iran	15 Parallel RCT 2 Cross-over RCT	3 Relaxation 3 Music-related 2 Resilience 5 MBSR 4 Yoga 1 Meditation 1 Aromatherapy	↑ Job satisfaction ↑ Quality of life ↑ General health ↓ Burnout ↓ Stress ↓ Anxiety ↓ Depression ↓ Fatigue	Yoga showed significant effect on burnout

Bischoff/ 2019/Germany	9 (1995–2017)	690 (9–282)	4 USA 1 China 1 Brazil 2 Sweden 1 Taiwan	7 RCT 1 Quasi 1 Pilot pre-post	4 Yoga 2 Physical exercise 1 Qigong 1 Tai Chi 1 Individually designed training	↓Emotional exhaustion ↓ Depersonalization ↓ Stress	Yoga and qigong can reduce stress among health personnel

Gillman/2015/Australia/	20 (1994–2013)	1811 (6–563)	8 USA 1 Australia 1 Italy 5 Canada 2 Sweden 1 Wales 1 Taiwan 1 Portugal	4 mixed 5 Grounded 3 Phenomenology 4 cross-sectional 1 concept mapping 2 Pre-post 1 RCT	2 Emotion-focused techniques 9 Stress management and coping 3 Compassion fatigues resilience strategies 2 Hoping and self-transcendence 2 Death education 2 Mentoring or buddy systems 1 Consultation	↑ Resilience ↑ Coping ↑ Job satisfaction ↑ Quality of care ↓ Stress ↓ Burnout	Many strategies can help nurses to cope with work challenges including strategies which promote team connection, help reduce stress and recovery, or help to deal with emotions from experiences

Ghawadra/2019/Malaysia	9 (2006–2017)	467	1 Canada 3 USA 1 Japan 1 Malaysia 1 Brazil 2 Portugal	2 RCT 3 QCT 4 Pre-post	7 MBSR 1 Mindful-gym 1 Self-related processing	↑ Job satisfaction ↑ Quality of life ↑ General health ↑ Relaxation ↑ Sense of coherence ↑ Self-compassion ↑ Serenity ↑ Empathy ↑ Mindfulness ↑ Happiness ↓ Burnout ↓ Stress ↓ Anxiety ↓ Depression ↓ Fatigue	MBSR can reduce burnout, stress, anxiety, depression, and fatigue, and increase job satisfaction, quality of life and so on among nurses

Otto/2021/Germany	6 (1997–2019)	716	2 Norway 2 Netherlands 1 Australia 1 USA	6 RCT	2 Comprehensive orientation training 2 Exercise 1 Positive psychology 1 Acceptance and commitment therapy (ACT) 1 Clinical lesson 1 Emotion training 1 supervision meeting 1 Stress management	↑ Job satisfaction ↑ Mental health ↑ Physical health ↓ Burnout ↓ Neck complaints	Cognitive-behavioral and multicomponent interventions can improve physical and mental health, job satisfaction, and can reduce burnout and neck complaints among elderly care nurses

Westermann/2014/Germany/	16 (2001–2012)	2253 (21–384)	3 Canada 1 Italy 3 Germany 3 Australia 1 Denmark 1 UK 2 USA 2 Netherlands	10 RCT 5 Quasi 1 Pre-post	13 Innovative caring strategies and communication skills for dementia 4 Mentoring or buddy systems 1 Stress coping 1 MBSR 1 Ergonomic and psychosocial training	↓ Low personal accomplishment ↑ Job satisfaction ↑ Intrinsic motivation ↓ Emotional exhaustion ↓ Depersonalization	Only a few interventions have positive influences on nursing staff burnout, we need more evidence to prove that can prevent burnout

Ciezar-Andersen/ 2021/Canada/	25 (1998–2019)	1778	10 India 8 USA	12 RCT 1 Quasi 9 Pre-post 2 Qualitative 1 Mixed	25 Yoga	↑ Coping in acutely stressful situations ↑ Psychiatric ↑ Physical health ↑ Self-compassion ↑ Self-care practices ↑ Quality of care ↑ Mindfulness ↑ Concentration ↓ Stress ↓ Anxiety ↓ Depression ↓ Burnout ↓ Musculoskeletal aches and pains	Yoga can improve mental and physical health among HHPs and HHP students

Aryankhes/2019/Iran	18 (12 for nurses) (2006–2017)	6055	2 UK 4 USA 4 Netherlands 1 Australia 1 Japan 1 Canada 2 Turkey 1 Iran 1 Israel 1 China	12 RCT 6 pretest-post-test	1 Thankful event 2 Electronic-mental health care 1 Consultation with physician 1 Participatory program 2 Yoga 1 Communication skill training 1 Professional program 1 Cognitive and emotive training 1 Psychosocial training 1 Coping skill 1 MBSR	↓ Emotional exhaustion ↓ Depersonalization	The interventions used to improve burnout were communication skills, teamwork, participatory programs, and psychological interventions such as Yoga, meditation, and MBRS

Lee/2016/Taiwan	7 (1998–2014)	766	2 USA 2 Netherlands 1 Canada 1 Spain	5 RCT 2 Quasi	1 Cognitive behaviors meeting 2 Coping and stress management 2 Refresher session 2 MBSR 1 Team-based supported 1 Cognitive coping strategies 1 Problem-solving	↓ Emotional exhaustion ↓ Depersonalization ↑ Low personal accomplishment	Coping strategies can reduce nurse burnout

Suleiman-Martos/2020/Canada	17 (2005–2019)	632 (13–91)	8 USA 2 Australia 1 Ireland 1 Brazil 1 Portugal 2 Canada 1 Iran 1 Japan	8 RCT 9 Quasi	17 MBSR	↑ Physical health ↑ Mental health ↑ Quality of care ↑ Resilience ↑ Life satisfaction ↑ Self-compassion ↑ Low personal accomplishment ↑ Job satisfaction ↓ Emotional exhaustion ↓ Burnout ↓ Stress ↓ Depression ↓ Depersonalization	MBSR and MBSCR can reduce burnout among nurses. However, it needs more evidence to prove it

**Table 2:** Quality appraisal of included systematic reviews using JBI (*n* = 11).

Citation	Q1	Q2	Q3	Q4	Q5	Q6	Q7	Q8	Q9	Q10	Q11	No. of questions met
[10]	O	O	O	X	O	O	O	O	N/A	O	O	9
[25]	O	O	O	X	O	O	O	O	N/A	O	O	9
[14]	O	X	U	O	O	O	O	O	N/A	O	O	8
[15]	O	O	O	O	O	O	O	O	N/A	O	O	10
[26]	O	O	O	O	O	O	O	O	N/A	O	O	10
[12]	O	O	O	O	O	O	O	O	N/A	O	O	10
[27]	O	X	O	O	O	O	O	O	N/A	O	O	9
[13]	O	O	O	X	O	O	O	O	O	O	O	10
[28]	O	X	O	O	X	X	O	O	N/A	O	O	7
[9]	O	X	O	O	O	O	O	O	N/A	O	O	9
[11]	O	O	O	X	O	O	O	O	N/A	O	O	9
% meeting criteria	100	63.6	90.9	63.6	90.9	90.9	100	100	9.1	100	100	Range 7–10

*Note.* Met appraisal question (O)/not meet appraisal question (X)/unclear (U)/not applicable (N/A); Question of checklist: (1) Is the review question clear and explicitly stated? (2) Were the inclusion criteria appropriate for the review question? (3) Was the search strategy appropriate? (4) Were the sources and resources used to search for studies adequate? (5) Were the criteria for appraising studies appropriate? (6) Was critical appraisal conducted by two or more reviewers independently? (7) Were there methods to minimize errors in data extraction? (8) Were the methods used to combine studies appropriate? (9) Was the likelihood of publication bias assessed? (10) Were recommendations for policy and/or practice supported by the reported data? (11) Were the specific directives for new research appropriate?

## Data Availability

Data are available upon request.

## Additional Points

*What Is Already Known about the Topic?* (1) Nurse burnout is a widespread and global problem. (2) Nurse burnout has been associated with negative individual and work-related outcomes. (3) Various strategies have been implemented to reduce nurse burnout. *What This Paper Adds.* (1) Strategies to reduce burnout can be categorized into three domains: mental health, physical activities, and professional competence. (2) The interventions most applied were mindfulness-based stress reduction, yoga, and competence education. (3) Individual-based strategies were shown to effectively eliminate emotional exhaustion, depersonalization, and occupational stress.

## References

[1] Woo T., Ho R., Tang A., Tam W. (2020). Global prevalence of burnout symptoms among nurses: a systematic review and meta-analysis. *Journal of Psychiatric Research*.

[2] Ghahramani S., Lankarani K. B., Yousefi M., Heydari K., Shahabi S., Azmand S. (2021). A systematic review and meta-analysis of burnout among healthcare workers during Covid-19. *Frontiers in Psychiatry*.

[3] Galanis P., Vraka I., Fragkou D., Bilali A., Kaitelidou D. (2021). Nurses’ burnout and associated risk factors during the COVID‐19 pandemic: a systematic review and meta‐analysis. *Journal of Advanced Nursing*.

[4] Tabanejad Z., Zareei M., Oskouie F., Ebadi A., Mesri M. (2022). Job burnout among nurses during COVID-19 pandemic: a systematic review. *Journal of Education and Health Promotion*.

[5] World Health Organization (Who) (2023). % of Nurses Working in Hospitals. https://gateway.euro.who.int/en/indicators/hfa_519-5330-of-nurses-working-in-hospitals/#id=19604.

[6] Jun J., Ojemeni M. M., Kalamani R., Tong J., Crecelius M. L. (2021). Relationship between nurse burnout, patient and organizational outcomes: systematic review. *International Journal of Nursing Studies*.

[7] Khatatbeh H., Pakai A., Al‐Dwaikat T. (2022). Nurses’ burnout and quality of life: a systematic review and critical analysis of measures used. *Nursing Open*.

[8] Dall’Ora C., Ball J., Reinius M., Griffiths P. (2020). Burnout in nursing: a theoretical review. *Human Resources for Health*.

[9] Aryankhesal A., Mohammadibakhsh R., Hamidi Y. (2019). Interventions on reducing burnout in physicians and nurses: a systematic review. *Medical Journal of the Islamic Republic of Iran*.

[10] Bischoff L. L., Otto A.-K., Hold C., Wollesen B. (2019). The effect of physical activity interventions on occupational stress for health personnel: a systematic review. *International Journal of Nursing Studies*.

[11] Lee H. F., Kuo C. C., Chien T. W., Wang Y. R. (2016). A meta-analysis of the effects of coping strategies on reducing nurse burnout. *Applied Nursing Research*.

[12] Otto D. (2021). Driven by emotions! The effect of attitudes on intention and behaviour regarding open educational resources (OER). *Journal of Interactive Media in Education*.

[13] Suleiman‐Martos N., Gomez‐Urquiza J. L., Aguayo‐Estremera R., Cañadas‐De La Fuente G. A., De La Fuente‐Solana E. I., Albendín‐García L. (2020). The effect of mindfulness training on burnout syndrome in nursing: a systematic review and meta‐analysis. *Journal of Advanced Nursing*.

[14] Ghawadra S. F., Abdullah K. L., Choo W. Y., Phang C. K. (2019). Mindfulness‐based stress reduction for psychological distress among nurses: a systematic review. *Journal of Clinical Nursing*.

[15] Gillman L., Adams J., Kovac R., Kilcullen A., House A., Doyle C. (2015). Strategies to promote coping and resilience in oncology and palliative care nurses caring for adult patients with malignancy: a comprehensive systematic review. *JBI Database of Systematic Reviews and Implementation Reports*.

[16] Clark S., Smidt J., Bauer B. (2018). Impact of therapy dog visits on outpatient nurse welfare and job satisfaction. *Pet Behaviour Science*.

[17] Grabbe L., Higgins M. K., Baird M., Craven P. A., San Fratello S. (2020). The Community Resiliency Model® to promote nurse well-being. *Nursing Outlook*.

[18] Montanari K. M., Bowe C. L., Chesak S. S., Cutshall S. M. (2019). Mindfulness: assessing the feasibility of a pilot intervention to reduce stress and burnout. *Journal of Holistic Nursing*.

[19] Higgins J. P. T., Thomas J., Chandler J. (2023). *Cochrane Handbook for Systematic Reviews of Interventions*.

[20] Fusar-Poli P., Radua J. (2018). Ten simple rules for conducting umbrella reviews. *Evidence-Based Mental Health*.

[21] Choi G. J., Kang H. (2022). The umbrella review: a useful strategy in the rain of evidence. *The Korean Journal of Pain*.

[22] Faulkner G., Fagan M. J., Lee J. (2022). Umbrella reviews (systematic review of reviews). *International Review of Sport and Exercise Psychology*.

[23] Aromataris E., Munn Z. (2020). Jbi manual for evidence synthesis. https://synthesismanual.jbi.global.

[24] Aromataris E., Fernandez R., Godfrey C. M., Holly C., Khalil H., Tungpunkom P. (2015). Summarizing systematic reviews: methodological development, conduct, and reporting of an umbrella review approach. *International Journal of Evidence-Based Healthcare*.

[25] Ciezar-Andersen S. D., Hayden K. A., King-Shier K. M. (2021). A systematic review of yoga interventions for helping health professionals and students. *Complementary Therapies in Medicine*.

[26] Jung S. E., Ha D. J., Park J. H. (2021). The effectiveness and safety of mind-body modalities for mental health of nurses in hospital setting: a systematic review. *International Journal of Environmental Research and Public Health*.

[27] Stuber F., Seifried-Dübon T., Rieger M. A. (2021). The effectiveness of health-oriented leadership interventions for the improvement of mental health of employees in the health care sector: a systematic review. *International Archives of Occupational and Environmental Health*.

[28] Westermann C., Kozak A., Harling M., Nienhaus A. (2014). Burnout intervention studies for inpatient elderly care nursing staff: systematic literature review. *International Journal of Nursing Studies*.

[29] Haslinda A., Tyng C. L. T. (2016). Job stress and coping mechanisms among nursing staff in a Malaysian private hospital. *International Journal of Academic Research in Business and Social Sciences*.

[30] Amin N. A., Quek K. F., Oxley J. A., Noah R., Nordin R. (2018). Emotional distress as a predictor of work-related musculoskeletal disorders in Malaysian nursing professionals. *The international Journal of Occupational and Environmental Medicine*.

[31] Kim Y., Khil J., Keum N., Keum N. (2022). The effects of mindfulness and Buddhist meditation coaching on mental health outcomes in college students. *Evidence-based Complementary and Alternative Medicine*.

[32] Dunn T. J., Dimolareva M. (2022). The effect of mindfulness-based interventions on immunity-related biomarkers: a comprehensive meta-analysis of randomised controlled trials. *Clinical Psychology Review*.

[33] Scheepers R. A., Emke H., Epstein R. M., Lombarts K. M. (2020). The impact of mindfulness‐based interventions on doctors’ well‐being and performance: a systematic review. *Medical Education*.

[34] Martínez J. P., Méndez I., Ruiz-Esteban C., Fernández-Sogorb A., García-Fernández J. M. (2020). Profiles of burnout, coping strategies and depressive symptomatology. *Frontiers in Psychology*.

[35] Janssen M., Heerkens Y., Kuijer W., Van Der Heijden B., Engels J. (2018). Effects of Mindfulness-Based Stress Reduction on employees’ mental health: a systematic review. *PLoS One*.

[36] Harold L. (2023). Health benefits of mindfulness-based stress reduction. https://www.verywellmind.com/benefits-of-mindfulness-based-stress-reduction-88861.

[37] Kiecolt-Glaser J. K., McGuire L., Robles T. F., Glaser R. (2002). Psychoneuroimmunology: psychological influences on immune function and health. *Journal of Consulting and Clinical Psychology*.

[38] Dutta A., Aruchunan M., Mukherjee A., Metri K. G., Ghosh K., Basu-Ray I. (2022). A comprehensive review of yoga research in 2020. *Journal of Integrative and Complementary Medicine*.

[39] Cramer H., Quinker D., Schumann D., Wardle J., Dobos G., Lauche R. (2019). Adverse effects of yoga: a national cross-sectional survey. *BMC Complementary and Alternative Medicine*.

[40] Shin S. (2021). Meta-analysis of the effect of yoga practice on physical fitness in the elderly. *International Journal of Environmental Research and Public Health*.

[41] Sivaramakrishnan D., Fitzsimons C., Kelly P. (2019). The effects of yoga compared to active and inactive controls on physical function and health related quality of life in older adults-systematic review and meta-analysis of randomised controlled trials. *International Journal of Behavioral Nutrition and Physical Activity*.

[42] Oldland E., Botti M., Hutchinson A. M., Redley B. (2020). A framework of nurses’ responsibilities for quality healthcare— exploration of content validity. *Collegian*.

[43] Pueyo-Garrigues M., Pardavila-Belio M. I., Canga-Armayor A., Esandi N., Alfaro-Díaz C., Canga-Armayor N. (2022). Nurses’ knowledge, skills and personal attributes for providing competent health education practice, and its influencing factors: a cross-sectional study. *Nurse Education in Practice*.

[44] Fukada M. (2018). Nursing competency: definition, structure and development. *Yonago Acta Medica*.

[45] Grande R. A. N., Berdida D. J. E., Villagracia H. N. (2022). The moderating effect of burnout on professionalism, values and competence of nurses in Saudi Arabia amidst the COVID‐19 pandemic: a structural equation modelling approach. *Journal of Nursing Management*.

[46] Aini N., Wihastuti T. A., Hariyanti T., Juwitasari J. (2023). Nurses’ burnout level and clinical competence in emergency department: a scoping review. *Jurnal Aisyah: Jurnal Ilmu Kesehatan*.

[47] Maghsoud F., Rezaei M., Asgarian F. S., Rassouli M. (2022). Workload and quality of nursing care: the mediating role of implicit rationing of nursing care, job satisfaction and emotional exhaustion by using structural equations modeling approach. *BMC Nursing*.

[48] Glawing C., Karlsson I., Kylin C., Nilsson J. (2024). Work‐related stress, stress reactions and coping strategies in ambulance nurses: a qualitative interview study. *Journal of Advanced Nursing*.

[49] Galaiya R., Kinross J., Arulampalam T. (2020). Factors associated with burnout syndrome in surgeons: a systematic review. *Annals of the Royal College of Surgeons of England*.

[50] Huang Z. P., Huang F., Liang Q. (2023). Socioeconomic factors, perceived stress, and social support effect on neonatal nurse burnout in China: a cross-sectional study. *BMC Nursing*.

[51] Zakeri M. A., Bazmandegan G., Ganjeh H. (2021). Is nurses’ clinical competence associated with their compassion satisfaction, burnout and secondary traumatic stress? A cross‐sectional study. *Nursing Open*.

[52] Cheng F., Meng A. F., Jin T. (2015). Correlation between burnout and professional value in Chinese oncology nurses: a questionnaire survey. *International Journal of Nursing Science*.

[53] Mlambo M., Silén C., McGrath C. (2021). Lifelong learning and nurses’ continuing professional development, a meta synthesis of the literature. *BMC Nursing*.

[54] Shin N., Choi Y. J. (2024). Professional quality of life, resilience, posttraumatic stress and leisure activity among intensive care unit nurses. *International Nursing Review*.

